# Transcriptomics–metabolomics joint analysis: New highlight into the triterpenoid saponin biosynthesis in quinoa (*Chenopodium quinoa* Willd.)

**DOI:** 10.3389/fpls.2022.964558

**Published:** 2022-10-19

**Authors:** Yulu Zhao, Yucong Ma, Jiawei Li, Bin Liu, Xiaoqing Liu, Jianheng Zhang, Min Zhang, Chunmei Wang, Liping Zhang, Wei Lv, Guojun Mu

**Affiliations:** ^1^ North China Key Laboratory for Crop Germplasm Resources of Education Ministry, Laboratory of Hebei Provincial Crop Germplasm Resources, Hebei Agricultural University, Baoding, China; ^2^ National Semi-arid Agricultural Engineering Technology Research Center, Shijiazhuang, China

**Keywords:** quinoa, triterpenoid saponins (TSs), triterpenoid biosynthesis, transcriptomics-metabolomics joint analysis, qRT-PCR

## Abstract

Quinoa (*Chenopodium quinoa* Willd.) contains various physiologically active substances, including vitamins, polyphenols, flavonoids, phytosterols, and saponins. Research showed that saponins were the protective substances in the outer layer of quinoa seeds to defend against microbes, herbivores, and insects. Because the aglycones of quinoa saponins are triterpenoids, they are called triterpenoid saponins (TSs). In addition, the presence of TS imparted bitterness in quinoa and resulted in anticancer and anti-inflammatory effects. In this study, the seeds of low-saponin quinoa, NT376-2 (N), and high-saponin quinoa, B-12071(B), at 30 and 60 days after flowering (DAF) were used to measure the TS content and evaluated for their transcriptomic and metabolomic profiles. The amounts of TS were found to significantly differ between all possible comparisons: N and B at 30 DAF (N1_*vs*_B1), N and B at 60 DAF (N2_*vs*_B2), N at 30 DAF and 60 DAF (N1_*vs*_N2), and B at 30 DAF and 60 DAF (B1_*vs*_B2). RNA sequencing (RNA-seq) was used to screen differentially expressed genes (DEGs) and revealed 14,703 upregulated DEGs and 26,267 downregulated DEGs in the four comparison groups. The 311 overlapping DEGs found in the four comparisons were used for Gene Ontology (GO) and Kyoto Encyclopedia of Genes and Genomes (KEGG) enrichment analyses to screen for DEGs related to TS biosynthesis in quinoa. Metabolomics analysis identified acetyl-CoA, 1-hydroxy-2-methyl-2-butenyl-4-diphosphate, farnesal, and (S)-2,3-epoxysqualene as the key differentially accumulated metabolites (DAMs). Transcriptomics–metabolomics joint analysis showed that triterpenoid biosynthesis and terpenoid backbone biosynthesis were the enriched pathways of TS biosynthesis; farnesal were the key DAMs shared in the four comparison groups and associated with 10 key candidate DEGs related to TS biosynthesis in quinoa. These results provided important references for in-depth research on the metabolic mechanism of TS in quinoa.

## Introduction

Quinoa (*Chenopodium quinoa* Willd., *2n* = *4x* = 36), an annual dicotyledonous plant belonging to the Amaranthaceae family, is known as “nutritional gold” and “superfood.” Currently, quinoa is used to cure obesity, cardiovascular diseases, diabetes, and other chronic diseases (Lim et al., 2020). Furthermore, research had shown that quinoa is affluent in all amino acids essential for human beings and contained various physiologically active substances, including vitamins, polyphenols, flavonoids, phytosterols, and saponins. Saponins were the major anti-nutritional factor in the grain and presented in the outer layer of quinoa seeds and were used to defend against microbes, herbivores, and insects in plants. El Hazzam et al. (2020) demonstrated that the quinoa saponin was the pentacyclic triterpenoid saponin (TS) derived from β-amyrin and was an important secondary metabolite of the triterpenoid biosynthesis. Research showed that the bitterness of quinoa was closely related to the presence of TS (Gómez-Caravaca et al., 2014). Nowadays, because TSs have diverse pharmacological activities such as hemolytic, anticancer, and anti-inflammatory, TS biosynthesis has been widely studied (Yao et al., 2020).

The TS biosynthetic pathway was mainly divided into three stages: the initial stage, the terpenoid framework construction stage, and the modification stage (Zhao et al., 2017). In the initial stage, 3-hydroxy-3-methylglutaryl-coenzyme A synthase (HMGS) catalyzes the condensation of acetoacetyl-CoA and acetyl-CoA to produce S-3-hydroxy-3-methylglutaryl-CoA (HMG-CoA). HMG-CoA and mevalonate kinase (MVK) *via* the mevalonic acid pathway (MVA) produce the upstream precursor isopentenyl diphosphate (IPP). The MVA pathway exists in the cytoplasm and endoplasmic reticulum and is the original pathway for triterpene and sterol biosynthesis (Huang et al., 2014). Meanwhile, the 2-C-methyl-D-erythritol-4-phosphate (MEP) pathway is also presented in the initial stage. Herein, IPP is synthesized by pyruvate through 1-deoxy-D-xylulose-5-phosphate synthase (DXS) to produce the isomer of IPP and dimethylallyl pyrophosphate (DMAPP). The MEP pathway, located in plastids, is mainly involved in the biosynthesis of diterpenes, monoterpenes, carotenoids, and isoprenes (Luo et al., 2016). In the terpenoid framework construction stage, IPP condenses with DMAPP to form farnesyl pyrophosphate (FPP) through geranyl pyrophosphate synthase (GGPS) and farnesyl-pyrophosphate synthase (FPS1). Subsequently, FPP forms the (S)-2,3-oxidosqualene under the action of squalene synthase (SQS1) and squalene epoxidase (SQE). (S)-2,3-oxidosqualene is an important precursor for the formation of the triterpenoid framework (Haralampidis et al., 2002). In the modification stage, the triterpenoid framework is hydroxylated by cytochrome P450 (CYP450) and is glycosylated by glycosyltransferases (UTG), which eventually forms TS.

At present, the research on TS is mainly presented in medicinal plants, such as *Panax notoginseng* and *American ginseng* (Niu, 2013). However, studies of TS in quinoa are mainly limited in its extraction methods. Ultrasonic extraction is the most common method for extracting TS and is beneficial to save the extraction times compared with the traditional extraction methods (Espinoza et al., 2021). Meanwhile, most research is also focused on the expression analysis of genes about TS in quinoa. Fiallos-Jurado et al. (2016) showed that candidate genes related to quinoa saponin biosynthesis, *Cqβ-AS*, *CqCYP716A78*, and *CqCYP716A79*, were induced by MeJA, suggesting that this phytohormone was used to modulate saponin biosynthesis in quinoa. So far, there were a few reports on the pathway of TS biosynthesis in quinoa. In addition, research revealed that TS shares a common precursor synthesis pathway with other triterpenoid compounds in the initial stage and the terpenoid framework construction stage; however, the modification stage is highly specific and diverse (Zhao and Li, 2018). Therefore, the TS biosynthesis pathway needs to be further explored. In this study, the interactions between the differentially expressed genes (DEGs) and differentially accumulated metabolites (DAMs) of TS biosynthesis in quinoa were investigated using transcriptomics–metabolomics joint analysis, and the key metabolic pathways of TS biosynthesis were explored. Results will provide an important reference for the in-depth study of biosynthetic mechanisms of TS in quinoa.

## Materials and methods

### Sample preparation

In this study, 140 quinoa germplasm materials from the National Semi-Arid Agricultural Engineering Science and Technology Research Center, Hebei Province, were used for the determination of saponin criteria (**Supplementary Table S1**). The saponin content between 4.7 and 11.3 mg/g was identified as a high-saponin quinoa variety, and the saponin content<0.2–0.4 mg/g was identified as a low-saponin quinoa variety (Ruiz et al., 2017). According to the classification criteria of saponin content in quinoa, the low-saponin quinoa variety, NT376-2 (N) (0.253 mg/g), and the high-saponin quinoa variety, B-12071 (B) (10.514 mg/g), were screened and were used for the next study. Significant differences of saponin content existed between the two quinoa varieties (*p*< 0.0001) (**Supplementary Figure S1**). N and B were planted separately at individual spacings of 18 cm and row spacings of 45 cm in Guyuan County, Hebei Province, P. R. China (41°31’21.59″N, 115°52’34.37″E, 1,422 m a.s.l.), in May 2020 and May 2021. The field was routinely managed, and the plants were labeled with tags after flowering from August to September 2021. Quinoa starts to flower at 40–55 days after sowing. Five grams of quinoa seeds for N and B at 30 DAF, 40 DAF, 50 DAF, and 60 DAF were collected and were used for measuring the content of TS with three biological replicates (**Supplementary Table S2**). Because the TS content of quinoa seeds at 30 DAF and 60 DAF was significantly different (*p*< 0.001), the 3–5 g of seeds of N at 30 DAF (N1), N at 60 DAF (N2), B at 30 DAF (B1), and B at 60 DAF (B2) were taken and frozen in liquid nitrogen and were used for the transcriptomics and metabolomics analyses.

### Extraction and measurement of triterpenoid saponins in quinoa

The TS content was measured using the vanillin-glacial acetic acid-perchloric acid colorimetric method with three biological replicates (Huang et al., 2019). In this study, 1-g seeds were grounded to powder at 40 Hz for 20 min by a high-speed grinder (FW100, China), mixed with ethanol (1:30), sonicated at 45°C for 20 min by Ultrasonic Cleaner (SB-5200DTDN, China) to collect the supernatant by centrifuge (SIGMA3-18K, Germany) at 3,000 rpm for 15 min. Subsequently, 0.2 ml sample solution was dried at 70°C for 15 min and heated with 0.2 ml 5% vanillin-glacial acetic acid solution and 0.8 ml perchloric acid solution at 70°C for 15 min. After the chromogenic reaction, the sample solution was cooled for 10 min and finally mixed with 4 ml glacial acetic acid for measurement of UV absorbance. The absorbance was measured at 545 nm using UV-Vis spectrophotometer (UV-5800, Shanghai, China) and compared with methanol as the control. The total content of TS in the sample solution was calculated as follows:


$${\text{m}}_{\text{total}}(\text{mg}/\text{g})=mV/Mv$$

Note: *m* is the mass of TS in the sample solution (mg) according to the calibration curve, *V* is the total volume of the sample solution (ml), M is the total mass of quinoa (g), and the *v* is the volume of the sample solution (ml). The two-tailed t-test was used to assess the significance of differences (*p_value*). In this study, “*,” “**,” and “***” mean *p*< 0.05, *p*< 0.01, and *p*< 0.001, respectively.

### Transcriptomics analyses

#### RNA sequencing and quality control

Total RNA was extracted from 12 samples of seeds (three biological replicates for N1, B1, N2, and B2) using TRIZOI Kit (Invitrogen, Carlsbad, CA, USA) (Saccenti et al., 2014). RNA quality was assessed on an Agilent 2100 Bioanalyzer (Agilent Technologies, Palo Alto, CA, USA) according to the manufacturer’s protocol, and RNA purity was tested using 1% agarose gel electrophoresis by NanoPhotometer spectrophotometer (NanoDrop Technologies, Wilmington, DE, USA). After total RNA was extracted, eukaryotic mRNA was enriched by Oligo(dT) beads, and the enriched mRNA was fragmented into short seqences using fragmentation buffer and reversly transcribed into cDNA using NEBNext Ultra RNA Library Prep Kit (NEB, USA). The cDNA libraries were sequenced using Illumina Novaseq6000 platform by Gene Denovo Biotechnology Co. (Guangzhou, China) and generated 150-bp paired-end reads. Raw reads containing 10% unknown nucleotides (N) or 50% low-quality bases (Q ≤ 20) were filtered with fastp default parameters (Chen et al., 2018) to get high-quality clean reads. Clean reads were aligned with the tetraploid quinoa reference genome (https://www.ncbi.nlm.nih.gov/genome/?term=txid63459[orgn]) by HISAT2 system (Kim et al., 2015) according to the bowtie2 method (Freeberg and Kim, 2016). ASM168347v1 was used as the proper assembly version.

### Identification of differentially expressed genes

The genes were counted, and the expression level of genes, Fragments Per Kilobase of exon model per Million mapped fragments (FPKM), was calculated by RSEM (Li and Dewey, 2011). Principal component analysis (PCA) was performed based on hypergeometric distribution, and the DEGs between different comparison groups (N1_*vs*_B1, N2_*vs*_B2, N1_*vs*_N2, and B1_*vs*_B2) were identified using the DESeq2 in the R package (Love et al., 2014). The |log2FC| ≥1 and false discovery rate (FDR)<0.05 were the criteria used to identify the significant DEGs in the transcriptomics, of which fold change (FC) denotes the difference in the relative expression levels of genes (Ruiying et al., 2020). FDR was obtained by correcting for the significance of differences (*p_value*) using the Benjamini–Hochberg method (Pertea et al., 2015). Venny 2.1 online web tool (http://bioinfogp.cnb.csic.es/tools/venny/) was used to create a Venn diagram to identify the intersectional genes among the above comparison groups.

### Gene Ontology and Kyoto Encyclopedia of Genes and Genomes enrichment analyses

The Gene Ontology (GO) and Kyoto Encyclopedia of Genes and Genomes (KEGG) enrichment analyses were performed based on the DAVID database (david.abcc.ncifcrf.gov/). GO enrichment analyses annotated DEGs from cellular components (CC), biological processes (BP), and molecular functions (MF) using GoSeq (Young et al., 2010). All DEGs were mapped with GO terms in the GO database (http://www.geneontology.org/), and gene numbers for each term were calculated. The *q_value* (Benjamini–Hochberg-corrected *p_value*)<0.05 was used as the threshold to screen the significantly enriched GO terms. KEGG enrichment analysis was applied to identify the significantly enriched pathways of DEGs, and the *q_value<*0.05 was used as the threshold (Kanehisa et al., 2004).

### Metabolomics analysis

#### Qualitative and quantitative analyses of metabolites

Liquid chromatography–tandem mass spectrometry (LC-MS/MS) was used for the qualitative and quantitative analyses of metabolites. Twelve freeze-dried seed samples (three biological replicates for N1, B1, N2, and B2) were extracted with 70% methanol at 4°C for 24 h, and the homogenate was centrifuged at 12,000 rpm for 10 min at 4°C. The supernatant was taken into a 2-ml centrifuge tube and was filtered through a 0.22-µm membrane to obtain the prepared sample for LC-MS. In this study, 20 µl was taken from each sample as the quality control (QC), and the remaining samples were used for LC-MS detection. The sample extracts were analyzed using ultraperformance liquid chromatography-electrosspray ionization mode-tandem mass spectrometry (UPLC-ESI-MS/MS) system. The UPLC conditions based on Thermo Ultimate 3000 system were set as f ollows: ACQUITY UPLC^®^ HSS T3 (150 mm × 2.1 mm, 1.8 µm, Waters) column, 40°C, 5 mM ammonium formate in water (A) and acetonitrile (B) at a flow rate of 0.25 ml/min. The injection volume of each sample was 2 μl after equilibration. The ESI-MS experiments were executed on the Thermo Q Exactive mass spectrometer with 3.8 kV and -2.5 kV spray voltage of positive and negative ion modes, respectively. The capillary temperature was 325°C (Zelena et al., 2009).

The format of raw data files was converted into mzXML format using Proteowizard (v3.0.8789) to differentiate the MS/MS data. The R (v3.3.2) package XCMS (Smith et al., 2006) was used to perform peak identification, peak filtration, and peak alignment for each metabolite to obtain the mass-to-charge ratio (m/z), retention time and intensity, and positive and negative precursor molecule. The identification of metabolites is based on the exact molecular formula (molecular formula error<20 ppm), and the peaks were matched with the mass spectrometry public databases, including HMDB (http://www.hmdb.ca), Massbank (http://www.massbank.jp/), LipidMaps (http://www.lipidmaps.org), and mzClound (https://www.mzcloud.org) to confirm the annotations for the metabolites.

### Identification of differentially accumulated metabolites

All detected metabolites were annotated on the MetWare database, and multivariate statistical processing of metabolomic data was performed by MetaboAnalyst version 5.0 software. Quality control was carried out by evaluating the repeatability in the process of sample extraction with three biological replicates. For a preliminary visualization of differences between different sample groups, the unsupervised dimensionality reduction method and the PCA were applied using R package models (http://www.r-project.org/). The orthogonal projection to latent structures-discriminant analysis (OPLS-DA) model was further validated by cross-validation and permutation test for the metabolomics data, and a score chart and permutation chart of each comparison group were drawn to visualize differences among the comparison groups. The variable importance in projection (VIP) score of the OPLS model was applied to rank the metabolites that were best distinguished among the four comparison groups (Saccenti et al., 2014). The VIP ≥1 and |log2 (fold change)| ≥ 2< 0.05 were used as the criteria to identify the DAMs. The identified DAMs were mapped with KEGG database to enrich the significant pathways based on the *q_value<*0.05.

### Transcriptomics and metabolomics joint analyses

To investigate the relationship of DEGs and DAMs annotated with KEGG pathways, the transcriptomics and metabolomics joint analyses were carried out using the Pearson correlation coefficient (PCC), and the PCC was calculated by the COR program in R language. The PCC ≥0.6 and ≥0.8 mean the normal or significant correlation between the DEGs and DAMs, respectively. The network diagram was visualized by Cytoscape software (Shannon et al., 2003). The DEGs and DAMs were annotated to the KEGG database to obtain their common KEGG pathway.

### Verification of differentially expressed genes by quantitative real-time PCR

qRT-PCR was used to verify the expression levels of the candidate DEGs. The total RNA was extracted from the 12 samples (three biological replicates of N1, B1, N2, and B2) and reverse-transcribed into cDNA for qRT-PCR. The ddH_2_O was added in the qRT-PCR reaction mixture, including 2 μl cDNA, 2 µl BlazeTaq™ SYBR ^®^ Green qPCR mix2.0 (Applied Biosystems, Carlsbad, CA, USA), 2 μl qPCR Primer (2 μM), and 2 μl cDNA Template, to make up to 20 μl. The qRT-PCR analysis was performed by Bio-Rad CFX96 qRT-PCR system, and the reaction conditions were set as follows: 95°C for 15 min, PCR cycle step at 94°C for 20 s, annealing, and extension step at 60°C for 34 s. The specific primers were designed using Premier 5.0 software based on the Coding sequence (CDS) sequence of the target gene (**Supplementary Table S3**). The *ACT7* in quinoa was used as the internal reference, and the calculation of relative expression of DEGs by the 2^−ΔΔCt^ method (Livak and Schmittgen, 2001) and t-test was applied to analyze the significant differences.

## Results

### Transcriptomics analyses

#### RNA sequencing and quality control analyses

Total RNA was extracted, and 12 cDNA libraries were constructed for high-throughput sequencing. After removing 10% of unknown nucleotides and 50% of low-quality data (Q20<20%), a total of 87.59 Gb of clean data were obtained. The Q20 and Q30 scores were 97.26% and 92.72%, respectively. The Guanine Cytosine (GC) content was maintained at 43.60%–44.83%. The N50 of each sample was more than 14,851 bp, indicating the high sequencing quality of each sample. The sequence comparison between the clean reads and the reference genome showed that more than 95.51% of clean reads were mapped to the reference genome, 12.49%–14.23% of clean reads were mapped to multiple locations in the reference genome, and 4.06%–4.59% of clean reads were unmapped to the reference genome, indicating that a high proportion of reads from each sample was compared with the reference genome (**Supplementary Table S4**). In addition, the PCA showed that the first and second principal components (PC1 and PC2) were 71.8% and 17.4% of the variation, and the samples of the same variety were clustered together and those of different varieties were apart. The correlation heatmap analysis showed that the Pearson correlation coefficient (PCC) ranged from 0.375 to 1, and most of these samples met with PCC >0.8, indicating that these samples were significantly different and have good biological repeatability (**Supplementary Figure S2**).

### Identification of differentially expressed genes and enrichment analyses

**Figure 1:**
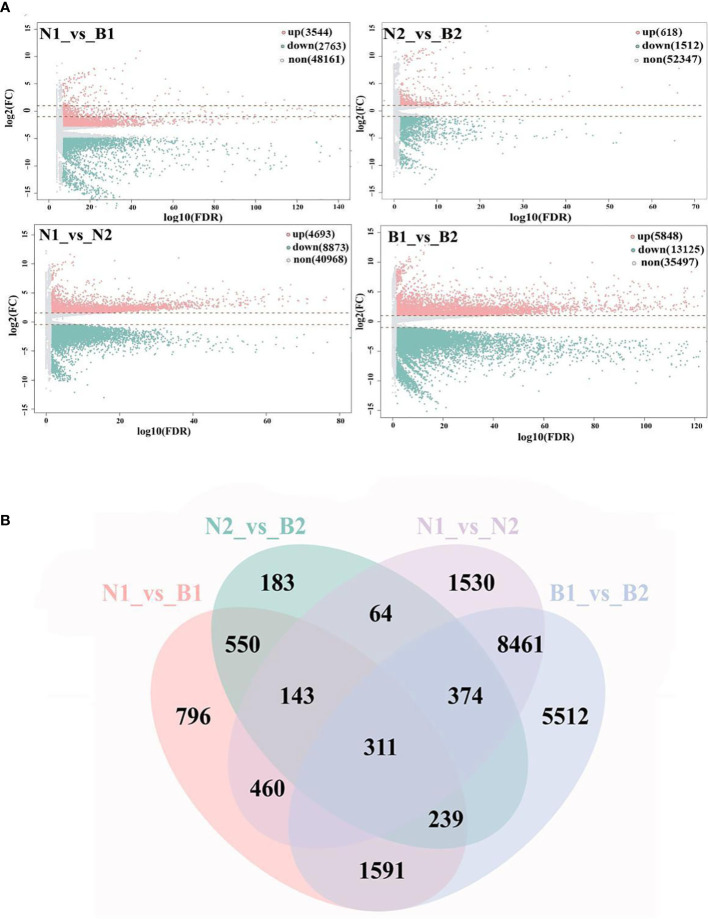
Screening and enrichment analyses of differentially expressed genes (DEGs). Volcano map **(A)** shows the DEGs identified from the N1_*vs*_B1, N2_*vs*_B2, N1_*vs*_N2, and B1_*vs*_B2. Up, down, and none in panel **(A)** mean the upregulated genes, downregulated genes, and no-expressed genes and are indicated in pink, green, and gray, respectively. The values at the top right indicate the number of upregulated, downregulated, and non-differential expression genes. The abscissa shows the false discovery rate (FDR) (-log10), and the ordinate shows the log2(FC). The Venn diagram **(B)** shows the overlapping of DEGs in the above four comparison groups. The numbers on the overlapping non-colored and colored parts indicate the DEGs in a single comparison group and the DEGs shared among different comparison groups.

'Base on |log2FC| ≥1 and FDR<0.05, a total of 40,970 DEGs were identified in the N1_*vs*_B1, N2_*vs*_B2, N1_*vs*_N2, and B1_*vs*_B2 comparison groups. Among these DEGs, 6,307 DEGs were detected in the N1_*vs*_B1, including 3,544 upregulated genes and 2,763 downregulated genes; 2,130 DEGs were detected in the N2_*vs*_B2, including 618 upregulated genes and 1,512 downregulated genes; 13,560 DEGs were detected in the N1_*vs*_N2, including 4,693 upregulated genes and 8,873 downregulated genes; and 18,973 DEGs were identified in the B1_*vs*_B2, including 5,848 upregulated genes and 13,125 downregulated genes (**Figure 1A**; **Supplementary Table S5**). Subsequently, Venn diagram analysis revealed that 311 overlapping DEGs were shared among the four comparison groups, whereas 796, 183, 1,530, and 5,512 were specific genes in each comparison group, respectively (**Figure 1B**). These results indicated that the numbers of DEGs in N1_*vs*_N2 and B1_*vs*_B2 were more than those in N1_*vs*_B1 and N2_*vs*_B2, and DEGs were mainly downregulated (except for N1_*vs*_B1).

To further investigate the functions of the identified DEGs, GO and KEGG enrichment analyses were adopted. The GO enrichment analyses showed that 1,742, 1,390, 2,257, and 2,452 GO terms were enriched in N1_*vs*_B1, N2_*vs*_B2, N1_*vs*_N2, and B1_*vs*_B2, respectively, and were annotated with three categories, including CC, BP, and MF. In the CC, the DEGs were mainly enriched in the cell (GO:0005623), cell part (GO:0044464), organelle (GO:0043226), and membrane (GO:0016020). In the BP, the DEGs were mainly enriched in the metabolic process (GO:0008152) and cellular process (GO:0009987). In the MF, the DEGs were mainly enriched in the catalytic activity (GO:0003824) and binding (GO:0005488) (**Supplementary Figure S3**; **Supplementary Table S6**). Moreover, the KEGG enrichment analyses indicated that 131, 109, 136, and 136 pathways were enriched in the above four comparison groups, respectively. Among these pathways, the DEGs were mainly enriched in metabolic pathways (ko01100) and biosynthesis of secondary metabolites (ko01110) (**Supplementary Figure S4**; **Supplementary Table S7**).

### Expression analysis of differentially expressed genes related to triterpenoid saponin biosynthesis

**Figure 2:**
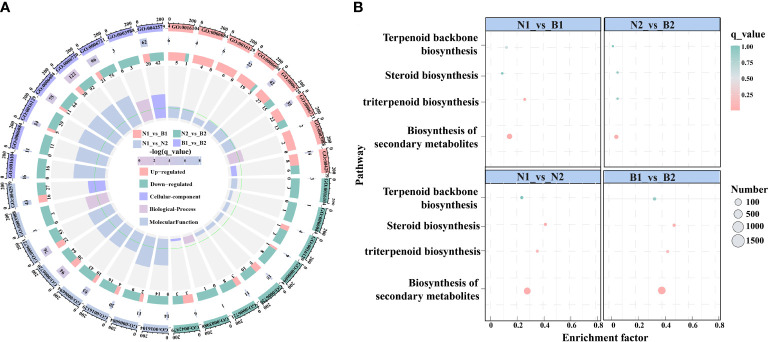
Enrichment analyses of the differentially expressed genes (DEGs) related to triterpenoid saponin (TS) biosynthesis. **(A)** shows Gene Ontology (GO) enrichment analyses of DEGs among the above four comparison groups. It is composed of four cycles. Cycle 1 (Cy1, the outermost cycle) means nine GO terms located in the above four comparison groups. Cycle 2 (Cy2, the secondary outer cycle) means the number of DEGs corresponding to GO terms. The different colors of the rectangles below the number are the *q_value* (-log10). Cycle 3 (Cy3, the sub-internal cycle) means the number of upregulated and downregulated genes corresponding to GO Classify2, which were labeled in pink and green, respectively. Cycle 4 (Cy4, the innermost cycle) means the enrichment range and the enrichment factors of GO terms, including cellular components, biological processes, and molecular functions. The green circular line indicates enrichment factor = 1. **(B)** shows the Kyoto Encyclopedia of Genes and Genomes (KEGG) pathway enrichment analyses of DEGs in N1_*vs*_B1, N2_*vs*_B2, N1_*vs*_N2, and B1_*vs*_B2 comparison groups. The abscissa and ordinate denote the enrichment factors and pathways, respectively. The size and color of the circles represent the number and *q_value* of DEGs.

In this study, there were 23 candidate DEGs related to TS biosynthesis, including two *CqHMGR*, one *CqHMG3*, two *CqHMGS*, one *CqGGPS*, two *CqMVK*, three *CqAAT1*, one *CqISPD*, one *CqDXS*, one *CqPMK*, one *CqFPS1*, three *CqSQS1*, one *CqSQE3*, two *CqSQE1*, and one *CqCYP716A15*, and one *Cqβ-AS* by Nr functional annotation (**Supplementary Table S8**). Subsequently, in the GO enrichment analyses, the TS biosynthesis-related DEGs were mainly annotated to the nine GO terms, including triterpenoid biosynthetic process (GO:0016104), acetyl-CoA-acetyltransferase activity process (GO:0006084), phytosteroid biosynthetic process (GO:0016129), steroid biosynthetic process (GO:0006694), isoprenoid metabolic process (GO:0006720), terpenoid metabolic process (GO:0006721), acetyl-CoA-acyltransferase activity (GO:0003988), transferase activity (GO:0046912), and microbody (GO:0042579). Based on these GO terms, the isoprenoid metabolic process was enriched in most DEGs, including 42, 15, 94, and 122 DEGs in N1_*vs*_B1, N2_*vs*_B2, N1_*vs*_N2, and B1_*vs*_B2, respectively (**Figure 2A**; **Supplementary Table S9**). In the KEGG pathway, the TS biosynthesis-related DEGs were mainly annotated with four pathways, consisting of biosynthesis of secondary metabolites (ko01110), triterpenoid biosynthesis (ko00909), terpenoid backbone biosynthesis (ko00900), and steroid biosynthesis (ko00100) (**Figure 2B**; **Supplementary Table S10**). The enrichment factors for the triterpenoid biosynthesis were 0.323, 0.077, 0.446, and 0.554, and the number of DEGs for terpenoid backbone biosynthesis was enriched with 18, 4, 36, and 52 DEGs in the four comparison groups, respectively. These results showed that isoprenoid metabolic process, triterpenoid biosynthesis, and terpenoid backbone biosynthesis were the key enrichment pathways related to TS biosynthesis in quinoa.

### Metabolomics analyses

#### Qualitative and quantitative analyses of metabolites

The off-targeted metabolomics analyses were used to perform the qualitative and quantitative analyses of metabolites in N1_*vs*_B1, N2_*vs*_B2, N1_*vs*_N2, and B1_*vs*_B2. The PCA plot of the detected metabolites revealed that the PC1 and PC2 explained 27.1% and 12.3% of the variation, respectively, indicating that the metabolites of the four comparison groups were changed significantly (**Supplementary Figure S5**). In addition, the result of OPLS-DA showed that Q2Y values in the four comparison groups were 0.919, 0.914, 0.978, and 0.972, respectively. The Q2 value was greater than 0.9, indicating that the model was stable and reliable (**Supplementary Figure S6**).

### Identification and enrichment analyses of differentially accumulated metabolites

A total of 9,940 DAMs in N1_*vs*_B1, N2_*vs*_B2, N1_*vs*_N2, and B1_*vs*_B2 were identified. There were 6,899, 4,369, 9,214, and 10,697 DAMs in each of the four comparison groups, respectively, among which 3,376, 2,621, 4,052, and 2,763 were upregulated metabolites, and 3,523, 1,748, 5,162, and 5,068 were downregulated metabolites, respectively (**Supplementary Table S11**). KEGG enrichment analysis revealed that 103, 106, 104, and 107 pathways were enriched in the four comparison groups, of which the identified DAMs were significantly enriched in biosynthesis of antibiotic (ko01130), biosynthesis of secondary metabolites (ko01110), glycerophospholipid metabolism (ko00564), and carotenoid biosynthesis (ko00906) (**Supplementary Figure S7**).

### Analyses of differentially accumulated metabolites related to triterpenoid saponin biosynthesis

**Figure 3:**
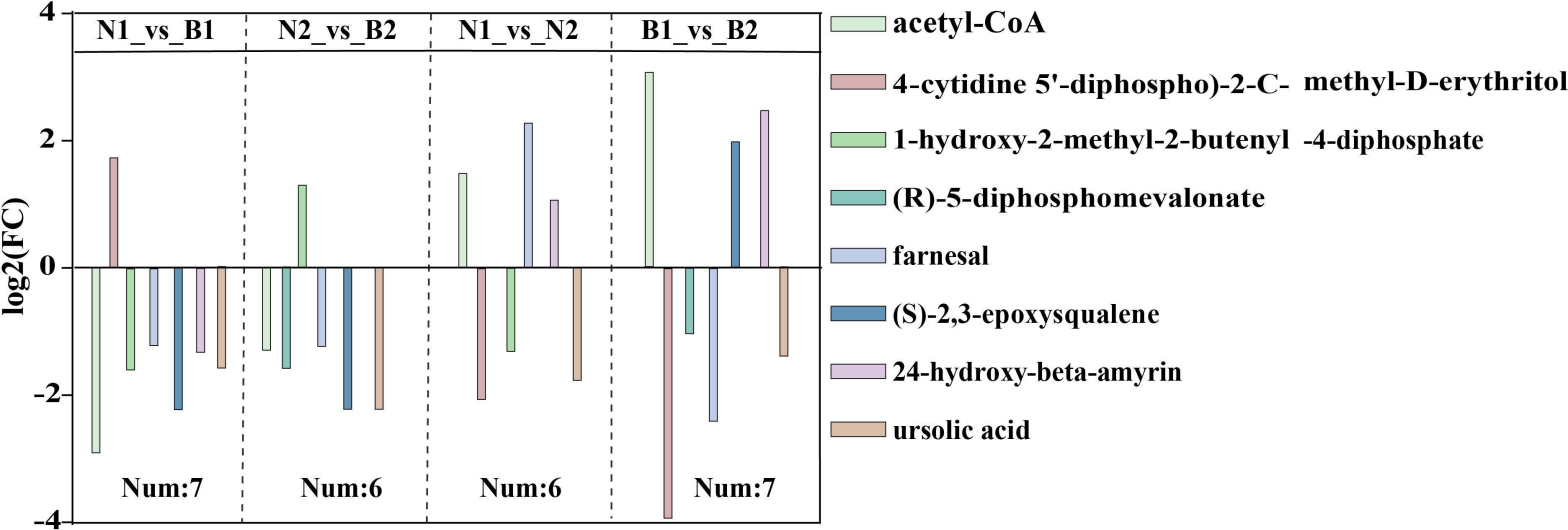
The differentially accumulated metabolites (DAMs) in the N1_*vs*_B1, N2_*vs*_B2, N1_*vs*_N2, and B1_*vs*_B2 comparison groups. Different colors in the legend indicate different types of DAMs. The ordinate indicates the abs (log2FC), and the number indicates the number of DAMs in the above four comparison groups. Rectangular squares represent the different comparison groups.

Among those identified DAMs, a total of eight DAMs related to TS biosynthesis were detected, including acetyl-CoA (POS_M810T467), 4-cytidine-5’-diphospho-2-C-methyl-D-erythritol (POS_M544T217), (R)-5-diphosphomevalonate (POS_M326T503), 1-hydroxy-2-methyl-2-butenyl-4-diphosphate (POS_M147T481), farnesal (POS_M221T594), (S)-2,3-epoxysqualene(POS_M409T832), 24-hydroxy-beta-amyrin (POS_M442T757), and ursolic acid (POS_M457T774) in N1_*vs*_B1, N2_*vs*_B2, N1_*vs*_N2, and B1_*vs*_B2 (**Figure 3**; **Supplementary Table S12**). The TS biosynthesis-related DAMs were involved in triterpenoid biosynthesis (ko00909) and terpenoid backbone biosynthesis (ko00900). These results indicated that the farnesal and acetyl-CoA were overlapped in all four comparison groups, and 1-hydroxy-2-methyl-2-butenyl-4-diphosphate and (S)-2,3-epoxysqualene were overlapped in three of the four comparison groups. Those DAMs was identified as the key DAMs based on the transcriptomics–metabolomics joint analysis.

### Transcriptomics–metabolomics joint analysis on triterpenoid saponin biosynthesis

**Figure 4:**
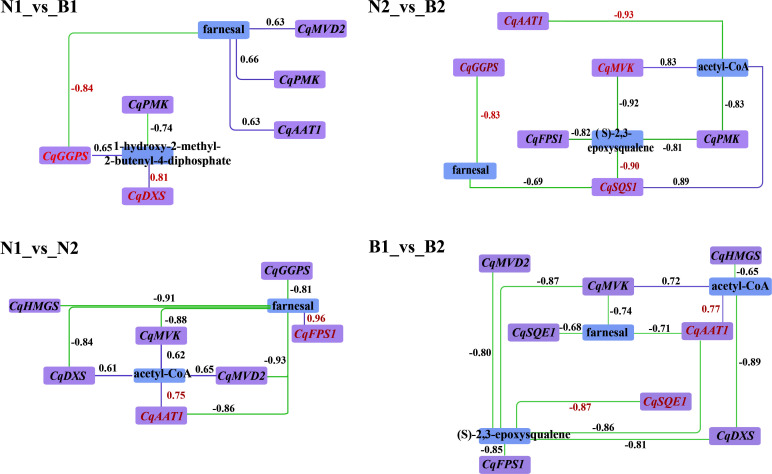
Correlation analysis between the candidate differentially expressed genes (DEGs) and differentially accumulated metabolites (DAMs). Purple and blue rectangles indicate the DEGs and DAMs associated with the biosynthesis of triterpenoid saponin (TS), respectively. The labels above the rectangle indicate the specific names of DEGs and DAMs. The purple solid lines and green dashed lines indicate positive correlation and negative correlation between DEGs and DAMs in the above four comparison. The numbers represent the correlation values, where the correlation coefficients and gene names are highlighted in red for the key DEGs and DAMs.

According to the DEGs and DAMs related to TS biosynthesis, the correlation analysis was conducted in the triterpenoid biosynthesis (ko00909) and terpenoid backbone biosynthesis (ko00900). In N1_*vs*_B1, seven DEGs were associated with farnesal and 1-hydroxy-2-methyl-2-butenyl-4-diphosphate, including two positively correlated DEGs and five negatively correlated DEGs. In N2_*vs*_B2, 13 DEGs were associated with farnesal, (S)-2, 3-epoxysqualene, and acteyl-CoA, including three positively correlated DEGs and 10 negatively correlated DEGs. In N1_*vs*_N2, 11 DEGs were associated with farnesal, including five positively correlated DEGs and six negatively correlated DEGs. In B1_*vs*_B2, 13 DEGs were negatively associated with farnesal and (S)-2,3-epoxysqualene. In summary, the result of transcriptomics–metabolomics joint analysis showed that farnesal was the DAM shared in the four comparison groups and correlated with 10 key candidate DEGs related to TS biosynthesis in quinoa, including *CqAAT1*, *CqHMGS*, *CqMVK*, *CqPMK*, *CqMVD2*, *CqDXS*, *CqGGPS*, *CqFPS1*, *CqSQS1*, and *CqSQE1* (*R^2^* ≥ 0.6) (**Figure 4**).

### Verification of differentially expressed genes related to triterpenoid saponin by qRT-PCR

**Figure 5:**
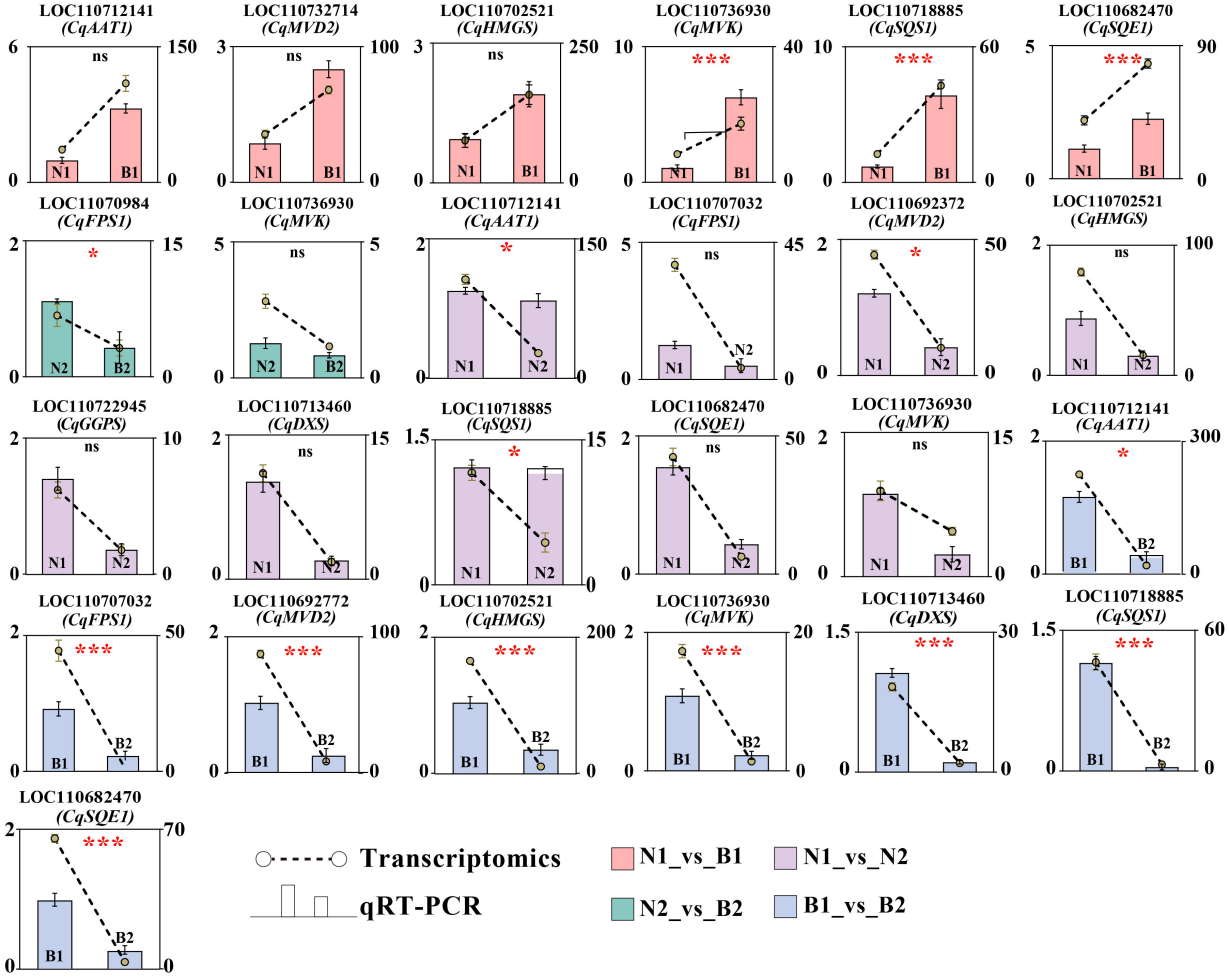
Verification of candidate differentially expressed genes (DEGs) by qRT-PCR. The Y-axis as the ordinate denotes the Fragments Per Kilobase of exon model per Million mapped fragments (FPKM) values. The ncbi serial number and name of the DEGs in N1_*vs*_B1, N2_*vs*_B2, N1_*vs*_N2, and B1_*vs*_B2 were marked in pink, green, purple, and blue, respectively. The error bars for qRT-PCR and transcriptomics were marked in black and yellow, respectively. In this figure, “ns,” “*,” and “***” mean *p* > 0.05, *p*< 0.05, and *p*< 0.001, respectively.

Ten key candidate DEGs related to TS biosynthesis in quinoa were screened by transcriptomics–metabolomics joint analysis and were validated by qRT-PCR detection. In N1_*vs*_B1, six DEGs were verified with upregulated genes, including *CqAAT1*, *CqMVD2*, *CqHMGS*, *CqMVK*, *CqSQS1*, and *CqSQE1*. In N2_*vs*_B2, two DEGs were verified with downregulated genes, including *CqMVK* and *CqFPS1*. In N1_*vs*_N2, 10 DEGs were verified with downregulated genes, including *CqAAT1*, *CqFPS1*, *CqMVD2*, *CqHMGS*, *CqGGPS*, *CqDXS*, *CqSQS1*, *CqSQE1*, and *CqMVK*. In B1_*vs*_B2, eight DEGs were verified with downregulated genes, including *CqAAT1*, *CqFPS1*, *CqHMGS*, *CqMVD2*, *CqMVK*, *CqDXS*, *CqSQS1*, and *CqSQE1*. These results show that *CqMVK* was validated in all of the four comparison groups, and that *CqAAT1*, *CqMVD2*, *CqHMGS*, *CqSQS1*, and *CqSQE1* were validated in three of the four comparison groups (**Figure 5**; **Supplementary Table S13**).

## Discussion

### Advantages of triterpenoid saponin content measurement and two quinoa varieties

TS imparts a bitter taste in quinoa, and their extracts can be processed into medicines and cosmetics. In this study, the TS content was measured by the vanilla-glacial acetic acid method because this method is available for the variety classification directly based on the color change, which plays an important role in the development of healthy products and TS drugs (Konishi et al., 2004). Previous studies have shown that the saponin content of high-saponin quinoa varieties is usually 4.7–11.3 mg/g and is called bitter quinoa, and the saponin content of low-saponin quinoa varieties is usually 0.2–0.4 mg/g, known as sweet quinoa (Ruiz et al., 2017). In this experiment, the saponin content classification of 140 quinoa varieties can provide a basis for future breeding work. Saponin was extracted from the selected high-saponin quinoa varieties, and the extract is developed into saponin drugs, and saponin was removed from low-saponin quinoa varieties to produce edible quinoa. In addition, although the taste of TS in quinoa was unpalatable, the bitterness of quinoa is beneficial for the cultivation of the plants, as saponins prevent herbivory. The TS content of 60 DAF was significantly higher than that of 30 DAF, which was in accordance with previous findings that the TS content in quinoa was increased along with kernel development (Mastebroek et al., 2000). This phenomenon has also been observed in *Panax ginseng* (Zhang et al., 2012) and *Panax notoginseng* (Wu, 2018).

### Regulation of key candidate differentially expressed genes and differentially accumulated metabolites associated with triterpenoid saponin biosynthesis

*HMGS* and *AAT1* are important catalytic genes in the MVA pathway (Luskey and Stevens, 1985; Dyer et al., 2009). It is supposed that overexpression of *HMGS* may lead to enhanced production of *Centellosides yield* in transgenic plants (Afroz et al., 2022). In this study, upregulation of *CqHMGS* promoted the accumulation of acetyl-CoA, and *CqAAT1* coregulated the synthesis of the upstream precursor IPP in the MVA pathway. This result infers that *CqAAT1* and *CqHMGS* promoted the accumulation of acetyl-CoA, thereby accumulating upstream substrates, and finally promoted the formation of downstream products of TS biosynthesis.

*MVK* is an important rate-limiting and ATP-dependent enzyme gene involved in TS biosynthesis (Bohlmann and Keeling, 2008). The increased expression of *NtMVK* improved the metabolite content in the MVA pathway in *Nicotiana tabacum* (Champenoy et al., 1999). In this study, *CqMVK* was upregulated at 30 DAF, while the relative content of (R)-5-diphosphomevalonate also increased. Thus, the upregulated expression of *CqMVK* could lead to the accumulation of (R)-5-diphosphomevalonate during the TS biosynthesis and consequently increased the TS biosynthesis. In addition, *DXS* is the first important rate-limiting enzyme gene in the MEP pathway (Rodríguez-Concepción et al., 2001). In *Camellia sinensis*, *CsDXS1* had different expression levels at different developmental stages (Guo et al., 2018). In this study, *CqDXS* was downregulated in both N1_*vs*_N2 and B1_*vs*_B2, indicating that its differential expression is associated with kernel development in quinoa.

**Figure 6:**
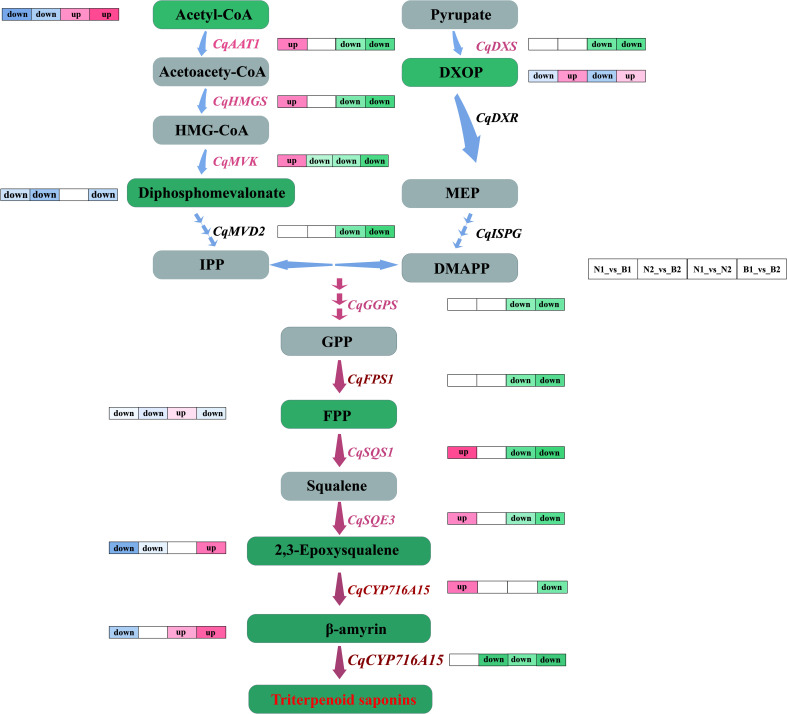
Flow-processing chart of the triterpenoid saponin (TS) biosynthesis in quinoa. The light blue and pink arrows between the metabolites indicate the upstream and downstream metabolic pathways. The labels next to the blue arrows are differentially expressed genes (DEGs), in which the key DEGs are labeled with pink. The gray and green rectangles represent the normal and key differentially accumulated metabolites (DAMs). The label “up” next to the genes and metabolites represents the upregulated key candidate DEGs and key DAMs in N1_vs_B1, N2_vs_B2, N1_vs_N2, and B1_vs_B2 comparison groups, and the label “down” next to the genes and metabolites represents the downregulated key candidate DEGs and key DAMs. The pathways marked by pink arrows are the key parts during the TS biosynthesis in quinoa.

Both *GGPS* and *FPS1* control the accumulation of TS and triterpenoid compounds together (Zhou et al., 2013; Zu et al., 2020). Previous research revealed that *SmGGPS* was significantly differentially expressed at different developmental stages in *Salvia miltiorrhiza* (Hua et al., 2014). In this study, *CqGGPS* and *CqFPS1* were upregulated at 30 DAF and downregulated at 60 DAF (**Figure 6**), providing the changes in gene expression levels along with the development of the kernels. Moreover, a unique mutual relationship between *TaGGPS* and *TaFPS1* was observed on identification and functional analyses of genes and metabolites in *Triticum aestivum* (Zhang, 2012). The result in this study showed that *CqGGPS* and *CqFPS1* were negatively correlated with farnesal, suggesting that these two genes play a negatively regulatory role in the TS biosynthesis in quinoa.

*SQS1* is a key enzyme gene that catalyzes the transformation of farnesal into TS and phytosterols (Shimada et al., 1998). *SQE3* is the regulatory enzyme gene involved in forming the TS framework (Laranjeira et al., 2015). In this study, *CqSQS1* and *CqSQE3* were significantly downregulated in N2_*vs*_B2, N1_*vs*_N2, and B1_*vs*_B2, which promoted the increase in the content of (S)-2, 3-epoxysqualene. It was speculated that *CqSQS1* and *CqSQE3* promoted the accumulation of TS by inhibiting its competing substrates, the phytosterols. This result is mutually verified with the research on *Saccharomyces cerevisiae* (Niu et al., 2012) and *Ashwagandha* (Singh et al., 2015). Meanwhile, *CqCYP716A15* is a key catalytic enzyme gene involved in the formation of TS, which oxidizes inert methyl groups of the triterpenoid framework (Nelson et al., 2008). In this study, the expression of *CqCYP716A15* was upregulated in both N and B at 30 DAF, but it was downregulated at 60 DAF, indicating that the expression of *CqCYP716A15* was regulated along with kernel development.

### Discoveries in the biosynthesis of triterpenoid saponin in quinoa

In this study, most DEGs were downregulated during the TS biosynthesis in N2_*vs*_B2, N1_*vs*_N2, and B1_*vs*_B2. Meanwhile, the expression levels of genes and their related metabolites were also significantly different in the developmental stages. Thus, we speculated that the DEGs in the TS biosynthesis mainly exist in the early developmental periods in quinoa kernels. *AsFPS1* was positively correlated with saponins in *Acanthopanax senticosus* (Zhou et al., 2013), and the upregulated expression of *FPS1* increased the content of saponin in *ginseng* (Kim et al., 2014). Interestingly, *CqFPS1* did not increase the content of farnesal during the biosynthesis of TS in quinoa in this study. On the contrary, a significant negative correlation was observed between *CqFPS1* and farnesal, implying that *CqFPS1* promoted TS by regulating other downstream genes during the TS biosynthesis. Furthermore, *CqCYP71615*, which hydroxylates the TS framework, was not annotated with any relevant metabolic pathway, although the Nr annotation provided that *CqCYP71615* was the key gene in β-amyrin synthesis and that the cytochrome P450 enzyme gene regulates the conversion from β-amyrin into TS. These data infer multiple regulation roles of *CqCYP71615* in the TS biosynthesis in quinoa (**Figure 6**).

## Data availability statement

The datasets presented in this study can be found in online repositories. The names of the repository/repositories and accession number(s) can be found below: https://www.ncbi.nlm.nih.gov/, PRJNA836289.

## Author contributions

GM, the corresponding author. GM and WL are responsible for the project planning and the material selection. YZ wrote the manuscript. YZ and YM performed most of the data integration, analysis, and prepared **Figures 1**, **2** and **3**. XL and JZ participated in most of experiment. BL, MZ, CW and LZ prepared **Figures 4**, **5** and **6**. JL revised the manuscript. All authors contributed to the article and approved the submitted version.

## Funding

This research was funded by the Key Project of Technologies for Industrialization of Quinoa in Bashang Region (19227527D), the Key Project of High-eficiency Technology Integration and Demonstration Project of Water-saving for Special Crops in Bashang Region of Hebei Province (21327005D), the Key Project of Modern Seed Industry Science and Technology Special Project of the Key Research and Development Program of Hebei Province (19226363D).

## Acknowledgments

We are specially appreciative to all members of National Semi-arid Agricultural Engineering Technology Research Center, Hebei Shijiazhuang, P.R.China for their efforts in breeding of varieties and selection of experimental materials. Thanks for the members of Quinoa Breeding Laboratory of Hebei Agricultural University, Baoding, P.R.China in helping to measure TS content and is grateful to omicsmart platform for the efforts in transcriptomics and Metabolomics sequencing.

## Conflict of interest

The authors declare that the research was conducted in the absence of any commercial or financial relationships that could be construed as a potential conflict of interest.

## Publisher’s note

All claims expressed in this article are solely those of the authors and do not necessarily represent those of their affiliated organizations, or those of the publisher, the editors and the reviewers. Any product that may be evaluated in this article, or claim that may be made by its manufacturer, is not guaranteed or endorsed by the publisher.
