# Supplementary figures and images for: Transcriptomics–metabolomics joint analysis: New highlight into the triterpenoid saponin biosynthesis in quinoa (Chenopodium quinoa Willd.)
Source: Front Plant Sci. 2022 Oct 19;13:964558. doi: 10.3389/fpls.2022.964558 (PMC9627512; doi:10.3389/fpls.2022.964558)

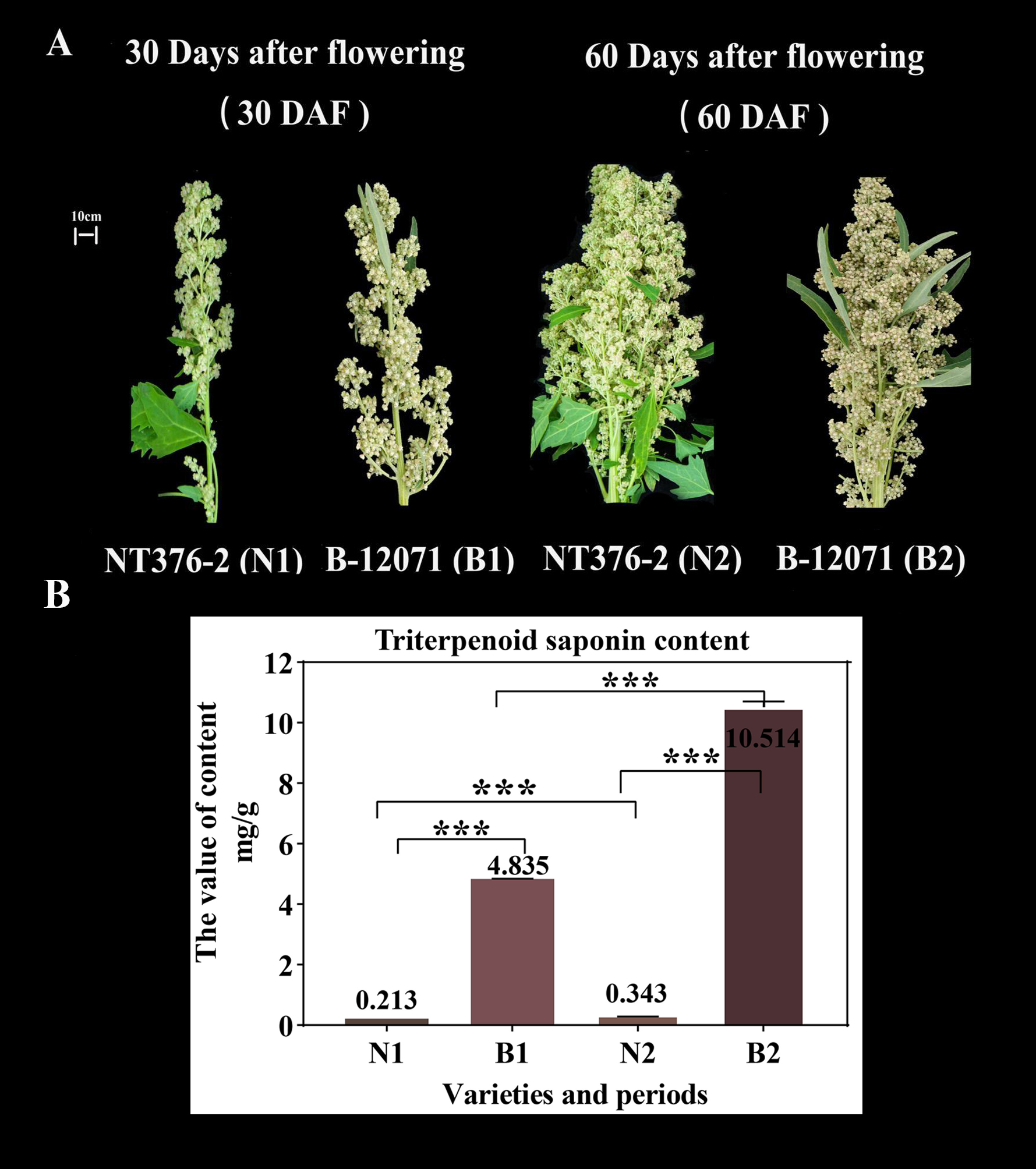

Supplement: Supplementary file 14 [file Image_1.jpeg]

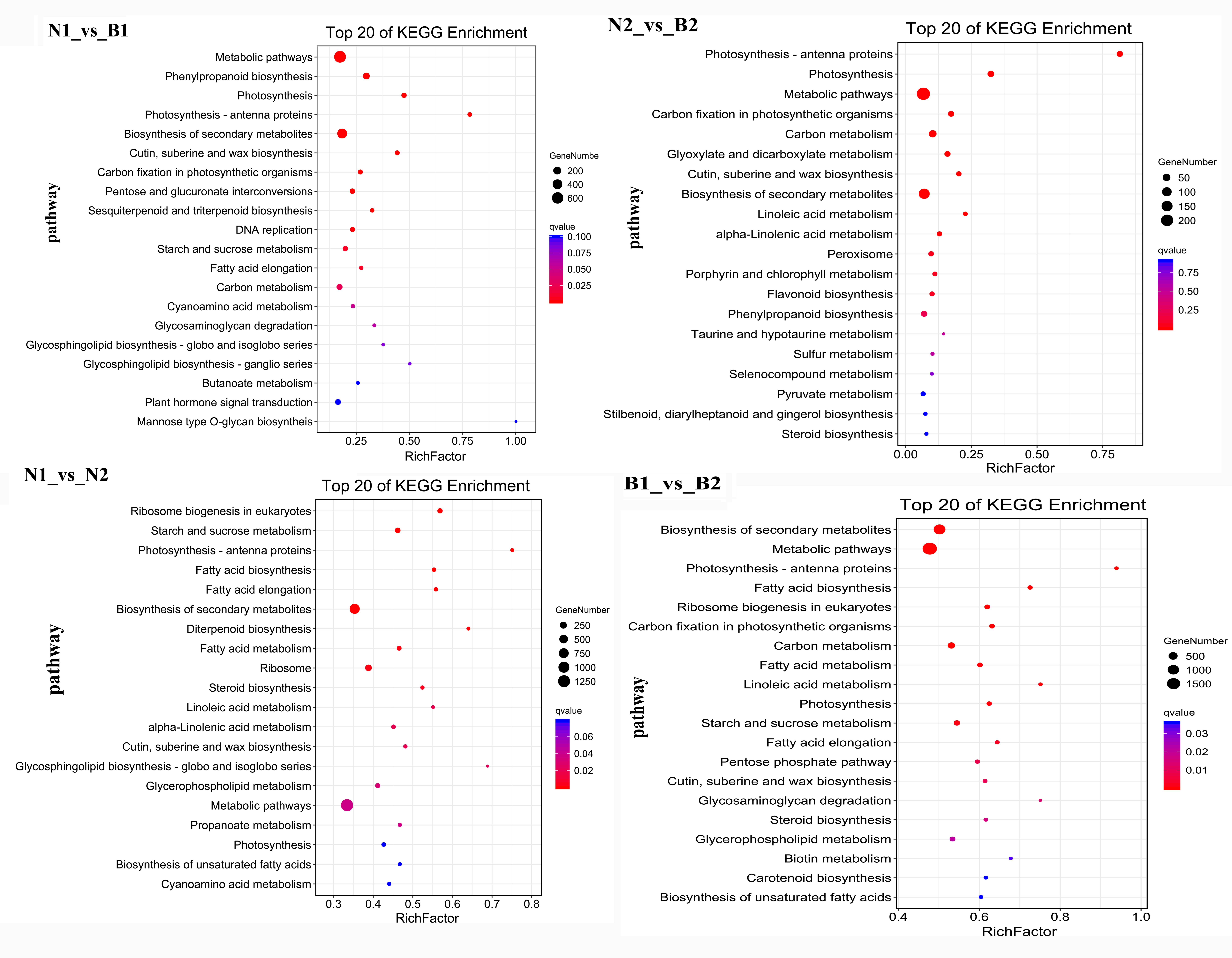

Supplement: Supplementary file 17 [file Image_4.jpeg]

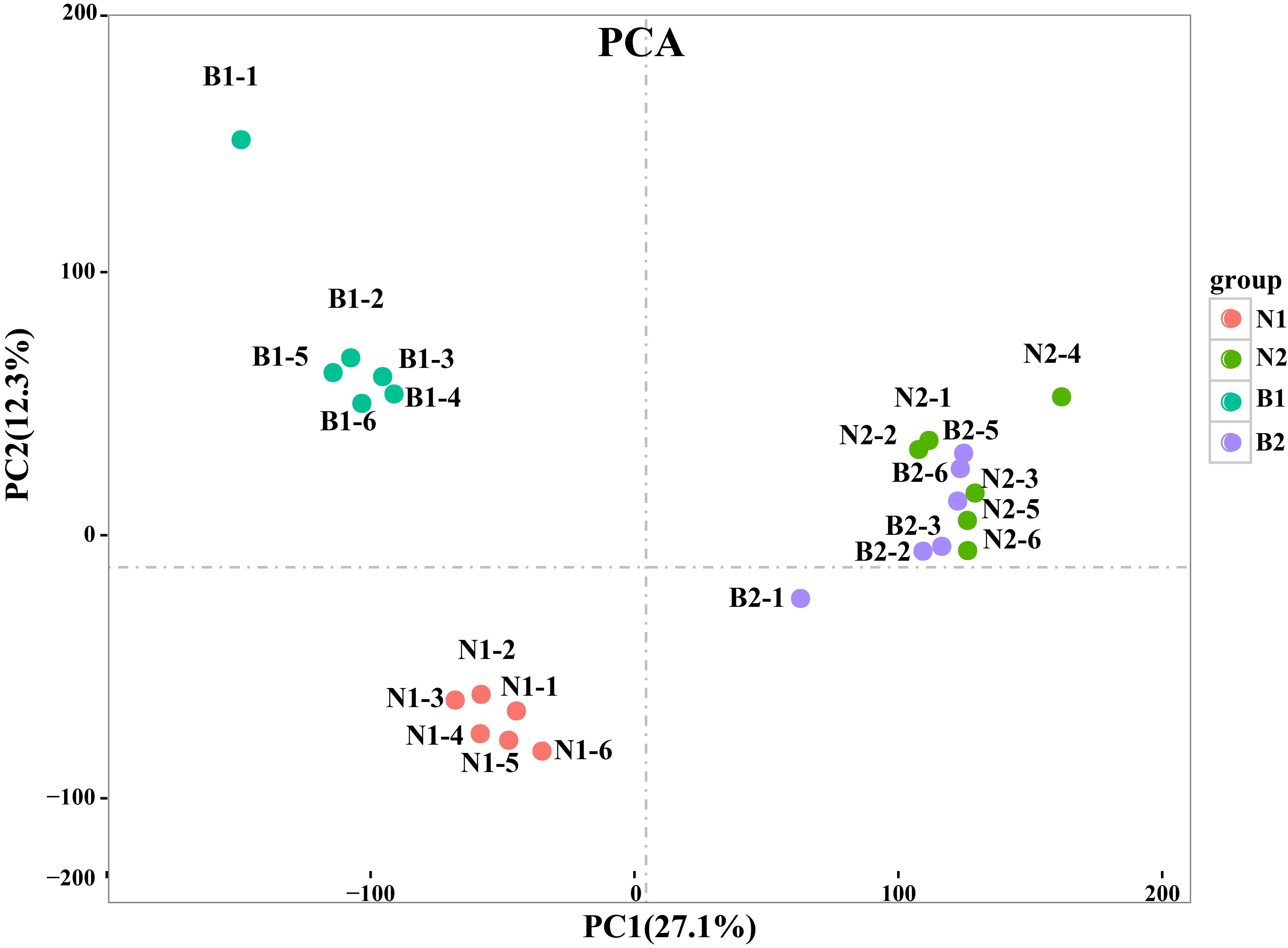

Supplement: Supplementary file 18 [file Image_5.jpeg]

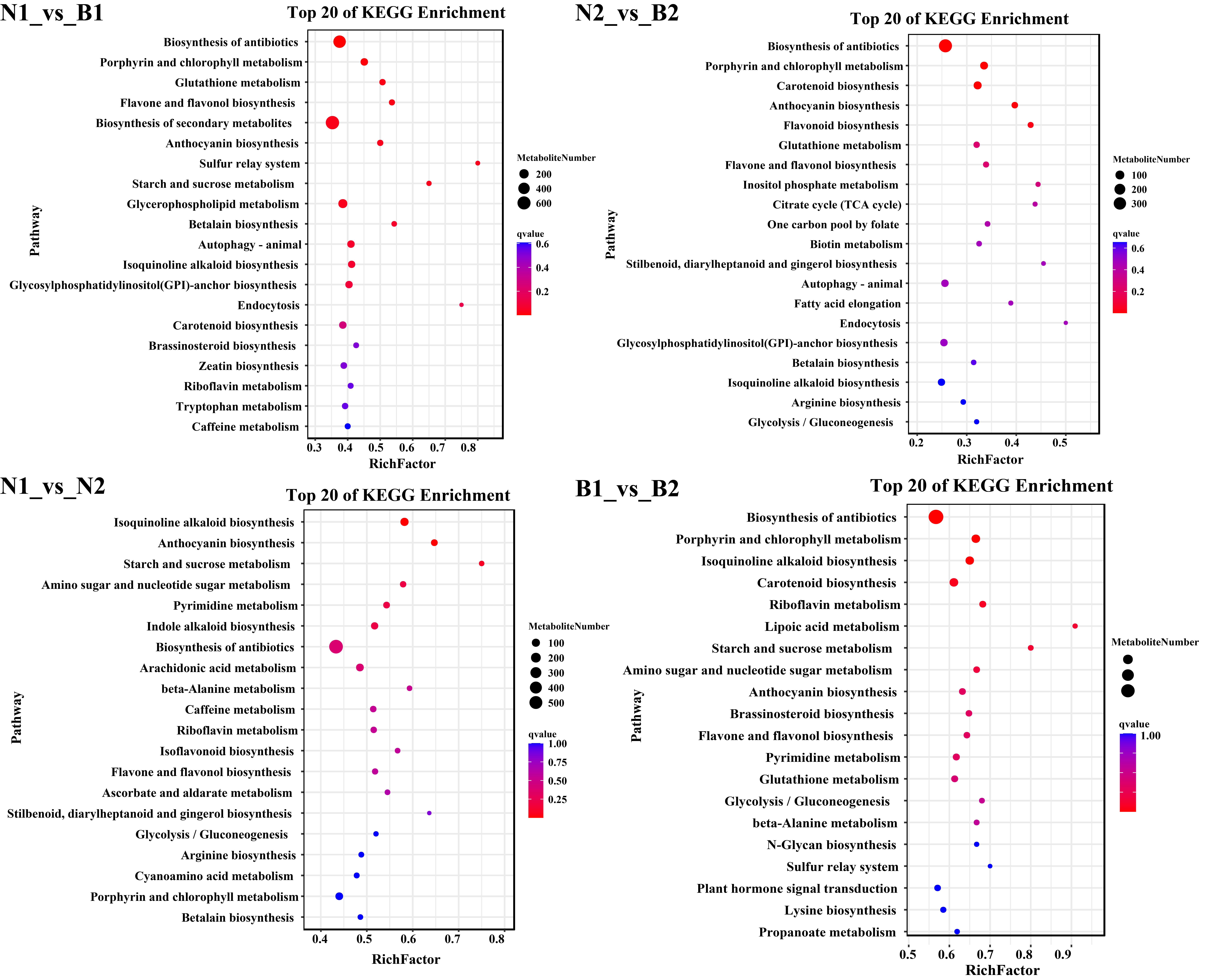

Supplement: Supplementary file 20 [file Image_7.jpeg]
